# Septic Arthritis Masquerading as a Flare of Rheumatoid Arthritis: A Not So Straightforward Diagnosis

**DOI:** 10.7759/cureus.18336

**Published:** 2021-09-27

**Authors:** Ramin Ahmad, Mejhorn Flash, Zekarias T Asnake, Joshua K Salabei, Matthew Calestino

**Affiliations:** 1 Internal Medicine, University of Central Florida College of Medicine, Graduate Medical Education/North Florida Regional Medical Center, Gainesville, USA

**Keywords:** rheumatoid arthritis, septic arthritis, synovial fluid analysis, fever, immunosuppression, polyarticular, pain

## Abstract

A typical presentation of septic arthritis (SA) includes pain, swelling, and erythema in the affected joint. Often, patients complain of inability to bear weight on the affected limb. However, some patients may present with subtle symptoms of pain and no fever or obvious swelling of the affected limb thus making the initial suspicion of SA low. Especially, patients with rheumatoid arthritis (RA) may present with polyarticular joint pain and initial synovial fluid analysis from an infected joint not consistent with overt septic arthritis. In such situations, the diagnosis of septic arthritis could be missed on delayed. In this case report, we present a 79-year-old female with a history of RA who presents with polyarticular pain, most notably in her right knee. SA was not initially suspected because of her history of RA and her current presentation with polyarticular pain. The initial synovial analysis did not suggest SA as well. However, cultures of synovial fluid from her right knee confirmed SA. Thus, we have highlighted that physicians should have a high suspicion for SA when addressing joint pain in RA patients.

## Introduction

Septic arthritis (SA) is an uncommon reason for visits to the emergency department (ED), though not rare, for example, in the United States in 2012, SA was responsible for 16,000 ED visits [1,2]. The mortality of SA is suggested to be about 11% for monoarticular involvement and as high as 50% for polyarticular involvement, therefore, SA is an orthopedic emergency warranting prompt diagnosis and treatment since its consequences are potentially devastating [3]. The higher mortality rate observed often correlates with comorbid conditions including advanced age, pre-existing joint disease, recent joint surgery or injection, skin or soft tissue infection, intravenous drug use, immunocompromised state (such as rheumatoid arthritis {RA}, diabetes, cirrhosis, HIV/AIDS), history of crystalline arthropathy, endocarditis, and recent bacteremia [1]. The most common presentation of SA is pain, swelling, and erythema in the affected joint. Patients often complain of worsening pain and inability to bear weight on the affected limb. However, some patients may present with subtle symptoms of pain and no fever or obvious swelling of the affected limb thus making the initial suspicion of SA low.

Patients with RA have additional risk factors for SA because, in addition to having damaged joints, they are often subjected to intraarticular injections to treat pain and are often on immunosuppressive medications. However, the diagnosis of SA in these patients can be easily missed because their symptoms of pain can be attributed to a flare of their RA. Thus, septic arthritis can be easily missed in patients with RA presenting with polyarticular pain because they may be mistakenly treated as a flare of RA.

In this case report, we present a 79-year-old female with a history of RA on immunosuppressive medications who presents with polyarticular pain, most notably in her right knee. She had received intraarticular analgesia to her right knee in an outpatient clinic a few days prior to presentation to the hospital. SA was not initially suspected because of her history of RA and her current presentation with polyarticular pain. Also, initial laboratory studies did not suggest SA. However, cultures of synovial fluid from her right knee confirmed SA. She subsequently underwent irrigation and debridement of her right knee and was discharged on long-term antibiotics without any complications.

## Case presentation

 A 79-year-old woman with a history of RA (taking leflunomide), hypertension, chronic kidney disease stage III, and paroxysmal atrial fibrillation presented to our hospital with worsening right knee pain with associated swelling and progressive inability to bear weight for about one week. She had pain in her other joints during this time but more so on her right knee. She also reported frequent flares of her RA managed outpatient by her primary care physician (PCP) and has been treated with intraarticular steroids and analgesics; she had just received an intraarticular injection of analgesics to her right knee about two days by her PCP prior to visiting the hospital. Her right knee pain was a constant 9 out of 10 ache that becomes sharp with any movement or touch. The pain was localized to the knee and was non-radiational. She also noted an increased amount of swelling to her knee during this time, limiting mobility. Her pain was only intermittently relieved with oxycodone. She denied paresthesias, fevers, and chills. Also denied any trauma to her knee, open wounds, recent dental procedures, or urinary symptoms. On initial examination, her vital signs were temperature of 97.9 degrees Fahrenheit, heart rate of 71 beats per minute, respirations of 17 per minute, blood pressure of 181/85 mmHg, and oxygen saturation of 100% on room air. Physical examination was unremarkable except for significant tenderness to light touch and limited active and passive range of motion, otherwise normal range of motion in other extremities. Pulses were equal and brisk in her bilateral extremities. Initial laboratory findings are shown in Table 1.

**Table 1:** Laboratory values on admission Thou: thousand; ESR: erythrocyte sedimentation rate; CRP: C-reactive protein

Parameters	Values	Reference values
White blood cell count	8.1	(4.5-11 thou/mm^3^)
Hemoglobin	10.2	(12.0-15.0 g/dL)
Hematocrit	31.3	(35.0-49.0%)
Platelet	265	150-450 thou/mm^3^)
Sodium	139	(136-145 mmol/L)
Potassium	3.5	(3.5-5.11 mmol/L)
Chloride	109	(98-107 mmol/L)
Bicarbonate	26	(21-32 meq/L)
Blood urea nitrogen	7.5	(7-18 mg/dL)
Creatinine	1.31	(0.60-1.30 mg/dL)
Glucose	90	(74-106 mg/dL)
Calcium	9.2	(8.5-10.1 mg/dL)
Phosphorus	2	(2.5-4.9 mg/dL)
Magnesium	1.9	(1.8-2.4 mg/dL)
Albumin	1.9	(3.5-5.0 g/dL)
ESR	71	(0-15 mm/h)
CRP	18.7	(0.00-0.29 mg/dL)

**Figure 1:**
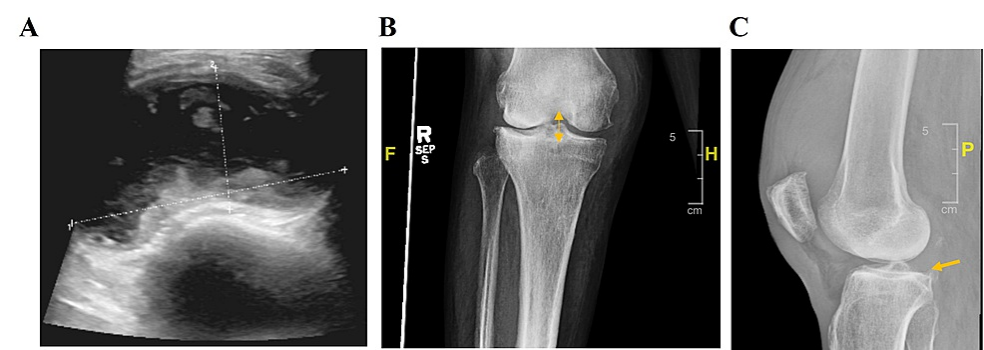
Representative images of the affected knee (A) Ultrasound of the right knee showing a complex large effusion measuring 12 × 7 × 3 cm (white lines). (B and C) Two view radiographs redemonstrating large effusion. Narrowing of the lateral tibiofemoral joint space (B: yellow double-headed arrow) and minimal articular surface bony spurring is noted in the patellofemoral and tibiofemoral joints (C: yellow arrow).

An ultrasound and two view radiographs of the right knee showed a large effusion (Figure 1). Blood and synovial fluid cultures were sent, and she was started on empiric treatment with ceftriaxone and continued on it despite a negative synovial fluid gram stain and profile not suggestive of an infectious etiology (Table 2, initial). The synovial fluid culture later came back positive for *Staphylococcus epidermidis *prompting antibiotic adjustment to cefazolin. Repeat right knee tap and culture were performed, given a high suspicion for contamination due to *Staphylococcus epidermidis*. Orthopedic surgery was consulted and they agreed with medical management while awaiting repeat synovial fluid analysis and culture. The repeat right knee analysis is shown in Table 2 (repeat) and repeat culture confirmed *Staphylococcus epidermidis* SA. The patient underwent emergent right knee irrigation and debridement without any complications. She felt significant symptom relief and was later discharged in stable conditions with home health services to continue treatment with cefazolin.

**Table 2:** Right knee synovial fluid analysis WBC: white blood cell count; RBC: red blood cell count; seg: segmented; thou: thousand

	Initial	Repeat
Fluid source	Right knee	Right knee
Fluid color	Yellow	Yellow
Fluid appearance	Turbid	Turbid
Fluid WBC (thou/mm^3^)	12,000	23,202
Fluid RBC (thou/mm^3^)	2500	2000
Fluid seg neutrophils (%)	93	87
Fluid lymphocytes (%)	0	2
Fluid monocyte/macrophages (%)	7	11
Fluid crystals	None seen	None seen

## Discussion

SA is most commonly caused by microbial seeding of a joint from underlying bacteremia, direct inoculation, or contiguous spread [4,5]. SA after intraarticular injection is uncommon; rarely it occurs in clusters due to unsafe injection practices [6]. SA can lead to irreversible joint destruction because of the action of proteolytic enzymes released from inflammatory cells and due to the ischemic environment caused by pressure on vessels from built-up exudative fluid in the joint space. Since cartilage is avascular and dependent on oxygen diffusion through the synovium, it is prone to destruction under such conditions.

Because of their inherent ability to bind connective tissue, most cases of septic arthritis are caused by Gram-positive bacteria. *Staphylococcus aureus* is the most common culprit. *Staphylococcus epidermidis*, on the other hand, can also cause SA especially after intraarticular joint injections in practices where sanitization protocols are laxed.

The diagnosis of SA combines clinical and laboratory findings. Classically, complaints of a tender, swollen, and painful joint with a positive synovial fluid analysis and culture is diagnostic. However, a definitive diagnosis can be made in a symptomatic patient in the setting of positive synovial fluid gram stain alone [7]. Also, a synovial fluid white blood cell counts >20,000 cells/microL with neutrophilic predominance is typical in SA. However, a cell count less than this has frequently been seen in patients with infections, especially in the immunocompromised or in arthritis due to disseminated gonococcal infection [1]. The synovial fluid white blood cell count is typically >50,000 cells/microL (and often >100,000 cells/microL) in most bacterial organisms, particularly SA caused by *Staphylococcus aureus*. Imaging studies can be helpful but cannot differentiate septic causes versus inflammatory arthritis [8]. Once the culprit organism has been identified with fluid cultures, therapy can be narrowed based on sensitivities. Generally, patients will require antibiotic treatment for weeks.

RA increases the risk for SA because of the presence of damaged joints and repeated intraarticular injections to treat pain. Many patients with RA and superimposed septic arthritis can present indolently (rather than acutely), often without fever or leukocytosis as seen in our patient. Conversely, RA itself may present with a "pseudoseptic arthritis" picture, including synovitis with a marked synovial fluid leukocytosis. Thus, diagnosing SA in patients with RA may not be a straightforward endeavor and thus calls for the execution of good diagnostic judgment on the part of the treating physician.

In the case of our patient, some distractors which could have swayed us from promptly diagnosing SA included: (1) the patient presented with polyarticular pain that could be mistaken for a flare of RA, (2) the patient was afebrile and non-toxic appearing, (3) initial synovial fluid analysis showed a negative Gram stain and leukocyte counts of <20,000 cells/microL, and (4) synovial fluid culture was positive for *Staphylococcus epidermidis* that could be attributed to contamination. However, our suspicion for SA remained high in this patient with risk factors and she was started on empiric antibiotic coverage immediately after the first synovial fluid was obtained.

Though *Staphylococcus epidermidis* SA is less common than SA caused by *Staphylococcus aureus*, it is likely that our patient’s joint was seeded during intraarticular injection in the outpatient. At the time of presentation to the hospital, she was likely at an early stage of SA with mild to moderate symptoms and initial synovial fluid analysis inconsistent with overt SA [9]. SA became more evident days later as shown by repeated synovial fluid analysis and symptoms persistence (Table 2).

## Conclusions

Herein, we have presented a case of SA in a patient with RA. Her presentation and initial laboratory studies were more consistent with inflammatory non-infectious arthritis; however, follow-up analysis and culture confirmed SA for which she received prompt treatment. This case highlights the high suspicion physicians should have for SA, especially in patients with risk factors such as RA who can have a septic joint but present with findings and symptoms more consistent with a flare of RA. In conclusion, we caution that intraarticular injections should be carefully administered under the best aseptic precautions especially in immunocompromised patients and that these patients should be followed promptly for septic arthritis if they present with any form of joint pain and effusion after intraarticular injection. 
